# VAMP5 Mediates Sevoflurane‐Induced Immune Modulation in Cervical Cancer: A Clinical Translational Study

**DOI:** 10.1155/anrp/9114246

**Published:** 2026-10-29

**Authors:** Zengzhen Zhang, Xianning Dong, Tingting Yu, Huiqing Li, Xinyu Yuan

**Affiliations:** ^1^ Department of Anesthesiology Shandong Provincial Third Hospital Shandong University Jinan Shandong 250031 China sdu.edu.cn; ^2^ Department of Pathology The Affiliated Hospital of Qingdao University Qingdao 266061 China qdu.edu.cn

**Keywords:** CD8^+^T-cells, cervical cancer, laparoscopic radical hysterectomy, sevoflurane, VAMP5

## Abstract

**Background:**

Sevoflurane, a commonly used inhaled anesthetic, has been implicated in modulating tumor biology and perioperative immune responses, yet the underlying molecular mechanisms remain poorly understood. This study aims to investigate whether sevoflurane regulates key immune‐related genes in cervical cancer patients undergoing laparoscopic radical hysterectomy (LRH) and to explore the clinical relevance of such regulation.

**Methods:**

Based on prior RNA‐seq screening, we established a two‐step validation framework to identify sevoflurane‐regulated genes in a clinical cohort. A total of 116 cervical cancer patients undergoing LRH were enrolled, including 70 who received sevoflurane anesthesia and 46 who received propofol anesthesia. Peripheral blood samples were collected at five perioperative time points. mRNA expression levels of candidate genes were measured by qRT‐PCR, lymphocyte subsets were analyzed by flow cytometry, and VAMP5 protein expression was assessed by intracellular staining. Bioinformatics analyses were performed using TCGA data to evaluate associations between candidate genes and tumor immune infiltration.

**Results:**

MAP3K7 and VAMP5 were validated as sevoflurane‐downregulated targets, showing sustained suppression postoperatively compared to the propofol group. VAMP5 expression correlated significantly with tumor progression and immune cell infiltration in cervical cancer, particularly CD8^+^ T‐cells, B‐cells, and macrophages. Among sevoflurane‐treated patients, those with lower VAMP5 expression exhibited delayed postoperative recovery of CD8^+^ T‐cell counts at 48 and 72 h after surgery, whereas no such difference was observed for other lymphocyte subsets.

**Conclusions:**

This study identify VAMP5 as a novel molecular link between anesthetic choice and immune modulation, suggesting that sevoflurane may influence perioperative immune recovery through vesicle‐associated mechanisms, with potential implications for optimizing anesthesia strategies to improve oncologic outcomes.

## 1. Introduction

Cervical cancer (CC) is a common malignant tumor that originates in the cervix of females [1]. Its pathogenesis is multifactorial, involving infectious agents such as human papillomavirus (HPV) [2], as well as complex interactions between genetic predisposition and environmental exposures [3]. For patients with early‐stage CC, surgical intervention remains the cornerstone of treatment [4].

Sevoflurane (Sev), a widely used inhalational anesthetic in clinical surgery, has recently attracted attention for its potential impact on tumor biology [5]. Several studies have suggested that Sev can influence the behavior of various cancer cells, including CC cells [6–8]. While most evidence points to its inhibitory effects on tumor cell proliferation, migration, and invasion [6], some reports have conversely indicated that Sev may create a microenvironment conducive to tumor progression [8]. This discrepancy may stem from the diverse molecular targets of Sev, which remain incompletely characterized.

Using RNA‐seq, we identified a series of regulated genes, many of which have previously been implicated in tumor development, suggesting that Sev may exert its functional effects through these candidate molecules [9]. The current study aims to investigate the clinical relevance of these candidate molecules in patients undergoing surgery for CC.

Laparoscopic radical hysterectomy (LRH) is a standard surgical technique for early‐stage CC, particularly in patients with FIGO Stage IA2 to IB1 [10]. Compared with open radical hysterectomy (ORH), LRH offers multiple advantages, including reduced surgical trauma, less intraoperative blood loss, faster recovery, and fewer postoperative complications [11]. Prior studies have shown that Sev anesthesia may modulate the immune microenvironment in patients undergoing LRH, potentially influencing oncologic outcomes [12]. However, the underlying molecular mechanisms remain unclear. This study seeks to determine whether the previously identified Sev targets mediate, at least in part, the immune‐modulatory effects of Sev anesthesia in patients with CC undergoing LRH.

## 2. Materials and Methods

### 2.1. Experimental Reagents

Experimental reagents were primarily used for molecular and cellular analyses. These included Ficoll‐Paque PLUS lymphocyte separation medium (Cat# 17144002) from Cytiva; TRIzol Reagent for RNA extraction (Cat# 15596026) from Thermo Fisher Scientific; the PrimeScript RT Reagent Kit (Cat# RR047A) and SYBR Green qPCR Master Mix (Cat# RR820A) from Takara Bio; fluorescently‐labeled antibody panels for flow cytometry from BioLegend, such as anti‐human CD3‐APC/Cy7 (Cat# 300318), CD4‐FITC (Cat# 317408), CD8‐PerCP/Cy5.5 (Cat# 301032), CD56‐PE/Cy7 (Cat# 362516), and CD19‐APC (Cat# 302212); and for VAMP5 protein intracellular staining, a rabbit anti‐human VAMP5 polyclonal antibody (Cat# PA5‐67597) from Thermo Fisher Scientific along with corresponding fluorescent secondary antibodies. All reagents were validated prior to use, and their selection and combination were tailored to enable precise analysis of immune cell phenotypes and the expression of specific target molecules in this study.

### 2.2. Bioinformatics Analysis

Bioinformatics analysis was performed using public databases. Based on data from the CC cohort in The Cancer Genome Atlas (TCGA), we employed the TIMER3 web tool (https://compbio.cn/timer3/) to analyze the correlation between candidate gene expression and tumor immune cell infiltration levels. Meanwhile, the UALCAN online platform (https://ualcan.path.uab.edu) was used to evaluate the association of these genes’ expression with clinicopathological features and patient prognosis.

### 2.3. Clinical Subjects

The study was conducted in accordance with the principles of the Declaration of Helsinki and was approved by the ethics committees of Shandong Provincial Third Hospital (lead site; approval number: KYLL‐2025053) and the Affiliated Hospital of Qingdao University. Informed consent was obtained from all patients. No individuals under the legal age of consent were included in this study. A total of 116 female patients diagnosed with CC and scheduled for LRH were enrolled. Among them, 70 patients received anesthesia induction and maintenance with Sev (Sev group), while 46 patients underwent anesthesia induction and maintenance with propofol, serving as the control group (Pro group). Major exclusion criteria included the presence of severe endocrine, immune, or circulatory system disorders; the use of immunomodulatory drugs or receipt of chemotherapy during the perioperative period, and the need for intraoperative blood transfusion.

### 2.4. Surgical Procedure

All patients underwent LRH with pelvic lymphadenectomy performed by experienced gynecologic oncologists. A standard four‐port transperitoneal approach was utilized. The procedure followed established oncologic principles: The round ligaments were ligated and divided, the retroperitoneum was opened to identify the ureters and dissect the paravesical and pararectal spaces, and the uterine arteries were isolated and ligated at their origin. The ureteral tunnels were dissected, and the cardinal and uterosacral ligaments were resected. The upper vagina was resected with a margin of at least 2 cm, and the specimen was removed en bloc via the vagina. Systematic pelvic lymphadenectomy concluded the procedure.

### 2.5. Anesthesia Management

Thirty minutes before induction, all patients received intramuscular premedication (0.1 g phenobarbital sodium and 0.5 mg atropine). Standard monitoring was established, followed by invasive arterial catheterization. Anesthesia was induced intravenously with sufentanil (0.3 µg/kg), midazolam (0.04 mg/kg), and propofol (2 mg/kg). After loss of consciousness, rocuronium (0.6 mg/kg) was administered to facilitate tracheal intubation.

Anesthesia was maintained with either continuous Sev inhalation (end‐tidal concentration 2%‐3%) in the Sev group or continuous propofol infusion (4–6 mg/kg/h) in the Pro group. Both groups received a continuous sufentanil infusion (0.15–0.35 µg/kg/h) for analgesia. Mechanical ventilation was initiated with a lung‐protective strategy: tidal volume of 6–8 mL/kg (predicted body weight) with a PEEP of 5–8 cm H_2_O. Pneumoperitoneum was established with CO_2_, maintaining an intra‐abdominal pressure of 12–14 mmHg. The patient was placed in a steep Trendelenburg position (20–30°). Ventilatory parameters were adjusted to maintain PetCO_2_ between 35 and 40 mmHg. Hemodynamics were closely monitored and managed with fluid or vasoactive drugs as needed. The depth of anesthesia was monitored using the BIS and maintained between 40 and 60.

### 2.6. Biomarkers Testing

Peripheral venous blood samples were collected at five time points: 30 min before anesthesia induction (T0); at the end of surgery (T1); and 24 (T2), 48 (T3), and 72 (T4) hours after surgery. Blood samples were processed by Ficoll density gradient centrifugation to isolate peripheral blood mononuclear cells (PBMCs). A portion of the cells was used for total RNA extraction to detect the mRNA expression level of the target genes via quantitative real‐time PCR (qRT‐PCR). Another portion of the cells was analyzed using multicolor flow cytometry: Firstly, lymphocyte subsets including total T‐cells, helper T‐cells, cytotoxic T‐cells, natural killer (NK) cells, and B‐cells were identified and counted based on surface markers (CD3, CD4, CD8, CD56, and CD19); subsequently, intracellular staining was performed to detect the expression intensity of VAMP5 protein within each lymphocyte subset, represented as mean fluorescence intensity (MFI).

### 2.7. Immunohistochemistry (IHC) Analysis

Tissue samples were fixed in 4% paraformaldehyde for 24 h at 4°C, paraffin‐embedded, and sectioned at 4 µm. Sections were deparaffinized, rehydrated, and subjected to antigen retrieval in citrate buffer (pH 6.0) by microwave heating for 15 min. Endogenous peroxidase and nonspecific binding were blocked with 3% H_2_O_2_ and 5% BSA, respectively. Sections were incubated with primary VAMP5 antibody (823,521, Zen‐Bioscience, China, 1:100) overnight at 4°C, followed by incubation with HRP‐conjugated secondary antibody (1:400) for 1 h at room temperature. Sections were then developed with DAB, counterstained with hematoxylin, dehydrated, and mounted. Images were captured under a light microscope with scale bars (100 µm). Using ImageJ, a consistent threshold was applied to define positive staining, and both the positive area percentage and mean optical density (IOD/area) were quantified per field.

### 2.8. Statistical Analysis

Student’s *t*‐test is used to compare differences between two groups of quantitative data. Repeated measures analysis of variance (ANOVA) was used to compare the trends of VAMP5 expression and lymphocyte subset counts over time between the two groups. Within the Sev group, patients were divided into high‐expression and low‐expression subgroups based on the median level of early postoperative VAMP5 expression. Analysis of covariance (ANCOVA) was employed to compare the differences in the magnitude of lymphocyte count decline between these two subgroups. A *p* value less than 0.05 was considered statistically significant.

## 3. Results

### 3.1. Analytical Framework for Validating RNA‐Seq‐Derived Gene Targets of Sev

Building upon our prior RNA‐seq screening which identified molecules potentially regulated by Sev in vitro [9], we established an analytical framework to validate these findings in a clinical cohort. The top candidates included 10 significantly downregulated genes (HERC1, MAP3K7, STAT1, HOXB2, KIF18A, EPS8, IKKB, POGK, VAMP5, and RSRC2) and ten upregulated genes (RASGRP, NFX1, CD40, PSME2, CTNNB1, C2CD5, TAGAP, MDC1, PTGS2, and RCHY1). To translate these in vitro results to a clinical context, we enrolled 116 surgical patients with CC, allocating 70 to the Sev group and 46 to the Pro group. The mRNA expression levels of these candidate genes in patient samples were quantified using qRT‐PCR. The groups were well‐balanced at baseline, with no significant differences in age, histologic type, ASA status, or FIGO stage (Table 1).

**TABLE 1:** Characteristics of cervical cancer patients.

Characteristics	Sevoflurane group (*n* = 70)		Propofol group (*n* = 46)		*p* value
	Number	%	Number	%	
Age					0.749
≤ 50	31	44.2	19	41.3	
> 50	39	55.7	27	58.7	
Histologic type					0.461
Squamous cell carcinoma	65	92.8	40	87.0	
Adenocarcinoma	5	7.1	6	13.0	
ASA status					0.629
I	41	58.6	29	63.0	
II	29	41.4	17	37.0	
FIGO stage					0.813
IA1‐IA2	32	45.7	20	43.5	
IB1	38	54.3	26	56.5	

The validation framework was applied as follows: (1) Baseline Equivalence: For each candidate gene, we first confirmed no statistically significant difference in expression between the Sev and Pro groups at baseline (T0). (2) Two‐Step Validation Criteria: A gene was considered clinically validated only if it met both sequential criteria: Criterion A (Temporal Change): In the Sev group, its expression was significantly altered (downregulated for down‐candidates and upregulated for up‐candidates) at one or more postoperative time points (T1–T4) compared to its own T0 level. Criterion 2 (Between‐Group Difference): At the same significant time points, its expression in the Sev group was also significantly different (lower for down‐candidates and higher for up‐candidates) compared to the Pro group. This framework ensured that validated targets demonstrated both a significant temporal change attributable to Sev and a specific between‐group difference, confirming Sev‐mediated regulation in the clinical setting (Figure 1).

**FIGURE 1:**
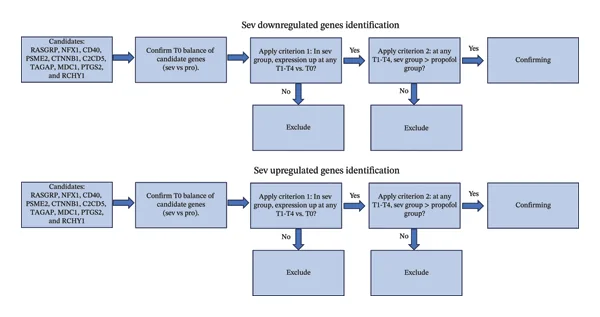
Clinical validation framework for candidate genes.

### 3.2. VAMP5 and MAP3K7 Are Validated as Sev‐Downregulated Targets

Following the aforementioned screening, we identified MAP3K7 and VAMP5 as Sev‐downregulated targets. As shown in Figure 2A, the mRNA expression of MAP3K7 in the Sev group decreased initially before recovering gradually from T0 to T4. Its expression was significantly lower than that in the Pro group at all postoperative time points (T1–T4), indicating sustained downregulation by Sev with slow recovery after anesthesia (Figure 2A). Similarly, VAMP5 mRNA expression exhibited a comparable Sev‐mediated suppression pattern. Notably, at the T4 time point, VAMP5 expression remained significantly lower in the Sev group compared to the Pro group, reflecting a more prolonged suppression after Sev withdrawal and suggesting that the regulatory effect of Sev on VAMP5 may persist longer (Figure 2B).

**FIGURE 2:**
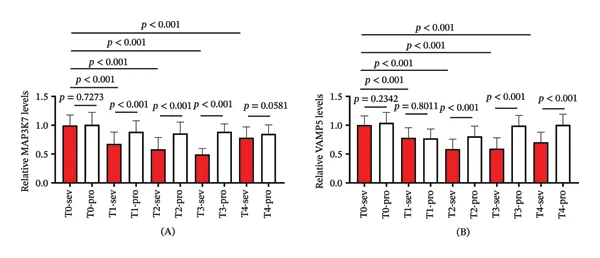
Comparison of (A) MAP3K7 and (B) VAMP5 mRNA expression between the Sev and Pro groups at the indicated time points.

### 3.3. Sev‐Regulated Genes Linked to Prognosis in CC

Subsequently, we employed the UALCAN platform to evaluate the association of MAP3K7 and VAMP5 expression with the CC prognosis (Figure 3A,B). Notably, VAMP5 expression levels showed a significant correlation with tumor progression (Figure 3B), while no statistically significant association was observed for MAP3K7 (Figure 3A).

**FIGURE 3:**
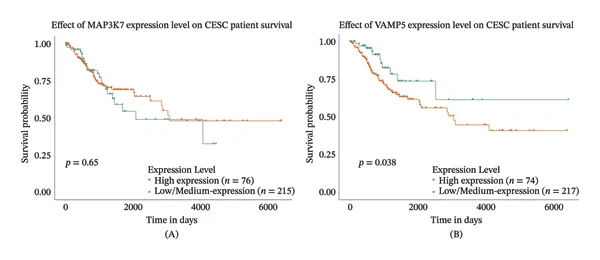
Effect of (A) MAP3K7 and (B) VAMP5 mRNA expression level on cervical squamous cell carcinoma and endocervical adenocarcinoma (CESC) patient survival based on the UALCAN analysis.

Further clinicopathological analysis confirmed that MAP3K7 expression was not significantly associated with the CC progression. No significant differences were found between tumor and nontumor tissues (Figure 4A), nor across different tumor stages (Figure 4B), histological grades (Figure 4C), or metastatic statuses (Figure 4D). In contrast, VAMP5 expression was significantly lower in tumor tissues compared to nontumor tissues (Figure 4E) and exhibited a further decrease in more advanced tumor stages (Figure 4F). However, no significant differences were detected across different grades or metastatic statuses (Figure 4G,H). Collectively, these findings highlight VAMP5 as a promising molecular target regulated by Sev in CC.

FIGURE 4MAP3K7 and VAMP5 mRNA expression levels in different cervical squamous cell carcinoma and endocervical adenocarcinoma (CESC) patients based on the UALCAN analysis. (A–D) Comparing MAP3K7 levels (A) between total CESC patients and normal controls; (B) among different cancer stages; (C) among different tumor grades; (D) among patients based on nodal metastasis status. (E–H) Comparing VAMP5 levels, (A) between total CESC patients and normal controls; (B) among different cancer stages; (C) among different tumor grades; (D) among patients based on nodal metastasis status.

**Figure:**
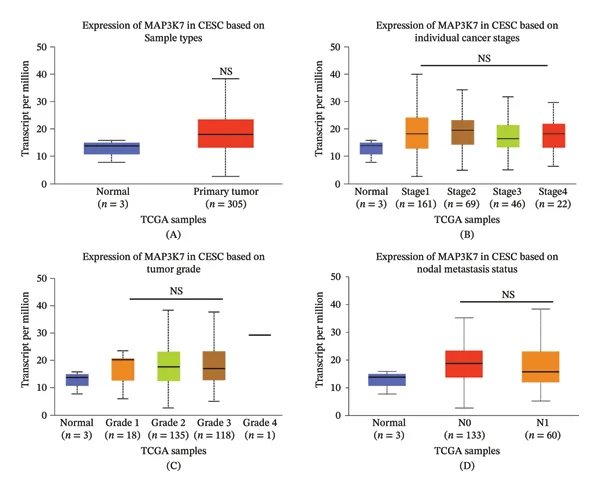

**Figure:**
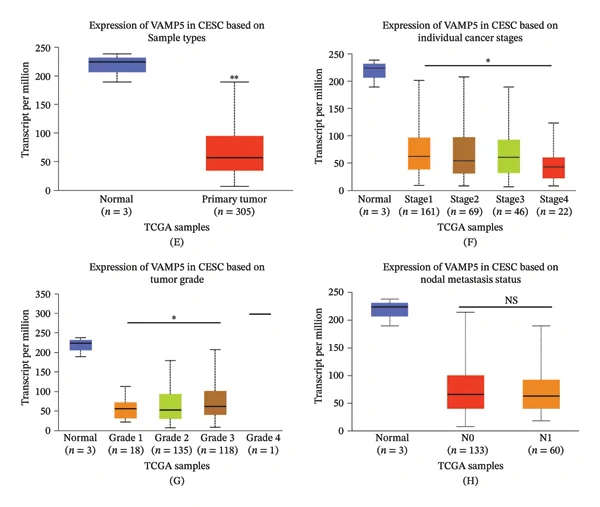

We next sought to validate these above findings in our own patient cohort. IHC on tumor tissues from the CC patient cohort in this study confirmed that VAMP5 levels were significantly downregulated in cancer tissues compared to adjacent noncancerous tissues (Figure 5A,B) and negatively correlated with FIGO stage (Figure 5C), corroborating the bioinformatics analysis and further supporting a role for VAMP5 in the CC progression.

**FIGURE 5:**
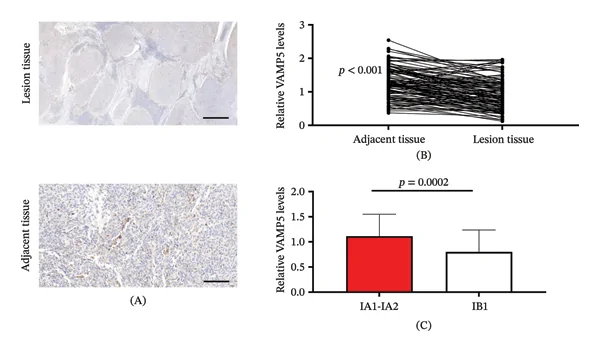
VAMP5 expressions were correlated with cervical cancer progression. (A, B) VAMP5 expression in tumor and adjacent normal tissues from 116 CC patients, assessed by immunohistochemistry (IHC). (A) Representative IHC micrographs showing VAMP5 immunostaining. Scale: 100 µm; (B) quantitative analysis of IHC staining intensity, comparing VAMP5 levels between tumor and adjacent tissues; (C) comparing the VAMP5 levels between IA1‐IA2 stage and IB1 stage.

### 3.4. Lymphocyte Subset Count Differences Between VAMP5^high^ and VAMP5^low^ Under Sev Group

Using TIMER3 online analysis tool, we found that in CC tissue samples, VAMP5 expression is closely associated with the infiltration levels of multiple immune cells, including CD4+ T‐cells, CD8+ T‐cells, B‐cells, and monocytes/macrophages (Figure 6). Therefore, we proceeded to explore the relationship between VAMP5 and immune cells in our clinical blood samples. We divided the Sev group patients (*n* = 70) into VAMP5‐high (*n* = 35) and VAMP5‐low (*n* = 35) subgroups and compared the dynamics of various immune cells in the blood across different time points. For all four measured immune cell types, regardless of VAMP5 expression level, their quantities showed a consistent pattern: a significant initial decrease from T0 to T4 followed by a gradual recovery (Figure 7A–D). Specifically for CD8+ T‐cells, a statistically significant difference was observed between the VAMP5‐high and VAMP5‐low groups at the T3 and T4 time points, but not at other time points (Figure 7A). For the other measured immune cells, no statistically significant differences were found between the two groups at any time point (Figure 7B,D).

**FIGURE 6:**
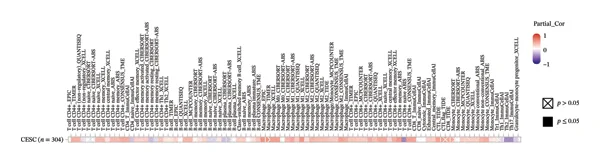
Correlation between VAMP5 expression and immune cell infiltration levels in cervical cancer tissues analyzed by TIMER3.

**FIGURE 7:**
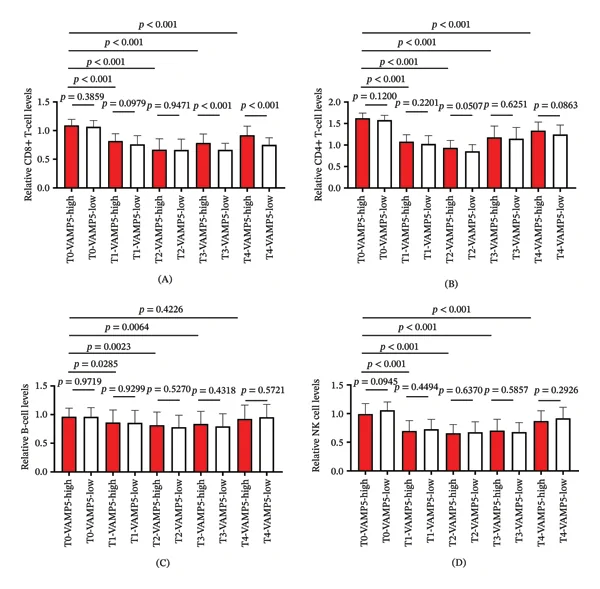
Dynamics of immune cell counts in blood from Sev group patients stratified by the VAMP5 expression level. Patients were divided into VAMP5‐high (*n* = 35) and VAMP5‐low (*n* = 35) subgroups. (A–D) The temporal changes in (A) CD8+ T‐cells, (B) CD4+ T‐cells, (C) B‐cells, and (D) natural killer (NK) cells, respectively, measured at different time points (T0 to T4).

## 4. Discussion

This study systematically evaluated the impact of the inhaled anesthetic Sev on postoperative immune responses in CC patients undergoing LRH by combining clinical trials with RNA sequencing technology, with a focus on its regulation of the key gene VAMP5. Compared to open surgery, LRH induces less traumatic inflammation and overall milder immunosuppression [13, 14]. This provided a relatively “clean” clinical background, facilitating the clearer isolation and observation of the specific immunomodulatory effects of the anesthetic itself, thereby avoiding the strong confounding effects of major surgical trauma.

The results demonstrated that Sev anesthesia significantly downregulated the expression of MAP3K7 and VAMP5 in patients postoperatively. The inhibition of MAP3K7 aligns with previously reported mechanisms by which Sev may influence macrophage polarization via the NF‐κB signaling pathway [9]. VAMP5, a key protein in vesicle fusion, not only regulates immune processes such as cytokine secretion and immune synapse formation [15, 16] but also shows aberrant expression in various cancers and correlates with the degree of immune cell infiltration [17]. This indicates that Sev regulates not ordinary “background genes” but core molecules directly involved in constructing and functioning within the tumor immune microenvironment. Therefore, this study may partially explain previous literature reports suggesting that Sev anesthesia can influence the tumor prognosis.

Further analysis revealed a close association between low VAMP5 expression and delayed postoperative recovery of CD8^+^ T‐cells in LRH patients under Sev anesthesia. The activation of CD8^+^ T‐cells depends on vesicle trafficking in T‐cell receptor signaling and the release of cytokines, such as IFN‐γ [18]. As a key regulator of vesicle transport, downregulation of VAMP5 may interfere with these processes, leading to impaired cytotoxic T‐cell function and delayed recovery. This finding carries direct clinical implications: In the era of minimally invasive surgery, the choice of anesthetic is no longer solely about intraoperative safety; it may become a new, adjustable “immune variable” directly impacting the speed and quality of a patient’s postoperative immune recovery. This provides a potential molecular basis for optimizing perioperative management strategies for LRH patients, suggesting that Sev’s effect on the tumor prognosis might be mediated through modulation of immune levels, particularly by exerting nontransient, profound effects on specific immune cells such as CD8^+^ T‐cells.

Recent studies identify VAMP5 as a broad‐spectrum antiviral factor that inhibits viral replication by interacting with viral NSP8 [19]. Given that persistent HPV infection drives cervical carcinogenesis, the VAMP5 downregulation observed in our tumor tissues may compromise intrinsic antiviral surveillance, potentially facilitating HPV persistence and malignant progression. This antiviral role may intersect with VAMP5’s immunomodulatory functions via vesicle trafficking, shaping the tumor microenvironment and CD8^+^ T‐cell responses. Although direct evidence linking VAMP5 to HPV restriction is lacking, our data suggest that Sev‐induced VAMP5 downregulation might affect both antiviral defense and immune recovery, warranting further investigation.

This study has limitations. The single‐center design and limited sample size may affect the generalizability of the results. The lack of long‐term follow‐up data also restricts our ability to assess the direct impact of these immune changes on patients’ ultimate clinical outcomes. Future research should expand the sample size, incorporate multicenter validation, and utilize functional experiments to elucidate the specific molecular mechanisms by which VAMP5 mediates Sev’s regulation of the immune microenvironment.

In summary, this study is the first to reveal, in CC patients treated with LRH, the clinical phenomenon where Sev anesthesia affects postoperative CD8^+^ T‐cell immune recovery through the downregulation of VAMP5. This expands the understanding of the role of anesthetic drugs in tumor immunomodulation and provides new scientific leads for promoting precision in perioperative anesthesia management to improve outcomes for cancer patients.

## Funding

This project was supported by a grant from the Shandong Province Medical and Health Technology Project (Project number: 202318000405).

## Conflicts of Interest

The authors declare no conflicts of interest.

## Data Availability

The datasets are available from the corresponding author on reasonable request.
